# Genome Sequences of Three Vaccine Strains and Two Wild-Type Canine Distemper Virus Strains from a Recent Disease Outbreak in South Africa

**DOI:** 10.1128/genomeA.00603-17

**Published:** 2017-07-06

**Authors:** Angelika K. Loots, Morné Du Plessis, Desiré Lee Dalton, Emily Mitchell, Estelle H. Venter

**Affiliations:** a National Zoological Gardens of South Africa, Gauteng, South Africa; b Department of Veterinary Tropical Diseases, Faculty of Veterinary Science, University of Pretoria, Onderstepoort, South Africa; c Department of Biotechnology, University of the Western Cape, Cape Town, South Africa; d Department of Zoology, University of Venda, Thohoyandou, South Africa; e College of Public Health, Medical and Veterinary Sciences, James Cook University, Townsville, Queensland, Australia

## Abstract

Canine distemper virus causes global multihost infectious disease. This report details complete genome sequences of three vaccine and two new wild-type strains. The wild-type strains belong to the South African lineage, and all three vaccine strains to the America 1 lineage. This constitutes the first genomic sequences of this virus from South Africa.

## GENOME ANNOUNCEMENT

Canine distemper virus (CDV) (family *Paramyxoviridae*) is an enveloped, nonsegmented, single-stranded, negative-sense RNA virus responsible for the disease canine distemper (CD), which has a high mortality rate in domestic and wild carnivore hosts (1–3). Vaccination has proven to be an effective intervention strategy against CDV in domestic hosts (3, 4); however, there is still a lack of quantitative data on the effects of a CDV vaccine in wildlife (5).

The RNA genome is 15,690 nucleotides (nt) long and consists of 6 genes that encode for a single envelope-associated protein [matrix (M)], 2 glycoproteins [the hemagglutinin (H) and fusion (F) proteins], 2 transcriptase-associated proteins [phosphoprotein (P) and large (L) protein], and a nucleocapsid (N) protein (6). The organization of the major genes was 3′-N-P-M-F-H-L-5′, each separated by untranslated regions (7). Only one serotype of CDV has been recognized, with several cocirculating genotypes based on H-gene variation clustering into the lineages America 1 and 2; Asia 1 and 2; Europe–South America 1; Europe wildlife; South America 2 and 3; Arctic; Rockborn-like; and Africa and Africa 2 (8–11).

Here, we report the complete genome of the three CDV strains (CDV_Buc, CDV_Nobi, and CDV_OVI) commonly found in vaccines and two wild-type strains (WT01 and WT02) from a recent CDV outbreak in South Africa. Both WT01 and WT02 were obtained from lung tissue collected from an infected African wild dog (*Lycaon pictus*), in Northern Cape Province, South Africa, and spotted hyena (*Crocuta crocut*), in Limpopo Province, South Africa, respectively. Samples were cultured and passaged between one and three times on VerodogSLAM cells grown in 25 cm^2^ tissue culture flasks (12). Viral RNA was extracted using Trizol (Invitrogen). Sequence-independent whole-genome reverse transcription-PCR amplification was used to prepare templates, which were sequenced on an Illumina MiSeq sequencer using the TruSeq sample preparation kit (Illumina). Data quality was assessed using FastQC version 0.11.2 software (http://www.bioinformatics.babraham.ac.uk). Paired sequence reads were analyzed with CLC Genomics Workbench version 6 (CLC bio, Aarhus, Denmark). Full-length genome sequences were assembled using a combination of mapping and *de novo* assembly.

The genomes of WT01, WT02, CDV_Buc, CDV_OVI, and CDV_Nobi are 15,690 nt, 15,649 nt, 15,673 nt, 15,670 nt, and 15,649 nt, respectively. Amino acid lengths of the six proteins encoded by each of the four genomes were 522 (N), 506 (P), 334 (M), 662 (F), 604 (H), and 2,183 (L). Multiple sequence alignments and Bayesian phylogenetic analysis according to the H-gene revealed that WT01 and WT02 belong to the South African lineage. The three vaccine strains were grouped in the America 1 lineage, which is consistent with other known vaccine strain groupings.

The two wild-type CDV strains constitute the first report of genomic sequences in South Africa. Data suggest the formulation of new and updated vaccines, considering the level of genetic variability obtained. These data contribute to the knowledge of CDV, which may be beneficial in determining effective preventative, diagnostic, and control measures for CD in South Africa.

### Accession number(s).

The genome sequences of WT01, WT02, CDV_Buc, CDV_Nobi, and CDV_OVI have been deposited in GenBank under the accession numbers KY971528, KY971532, KY971529, KY971530, and KY971531, respectively.
